# Effects of Marine-Derived Components on Cardiovascular Disease Risk Factors and Gut Microbiota Diversity

**DOI:** 10.3390/md22110523

**Published:** 2024-11-20

**Authors:** Ingrid Lamminpää, Amedeo Amedei, Cinzia Parolini

**Affiliations:** 1 Department of Clinical and Experimental Medicine, University of Florence, 50134 Florence, Italy; ingrid.lamminpaa@unifi.it; 2 SOD of Interdisciplinary Internal Medicine, Azienda Ospedaliera Universitaria Careggi (AOUC), 50134 Florence, Italy; 3 Network of Immunity in Infection, Malignancy and Autoimmunity (NIIMA), Universal Scientific Education and Research Network (USERN), 50134 Florence, Italy; 4 Department of Pharmacological and Biomolecular Sciences, ‘Rodolfo Paoletti’, Via Balzaretti 9, Università degli Studi di Milano, 20133 Milano, Italy

**Keywords:** cardiovascular diseases, atherosclerosis risk factors, microbiota, fish protein hydrolysates, seaweeds, marine-derived ingredients, probiotics

## Abstract

Cardiovascular diseases (CVDs), which comprise coronary heart disease, hypertension, and stroke, collectively represent the number one cause of death globally. Atherosclerosis is the dominant cause of CVDs, and its risk factors are elevated levels of low-density lipoprotein cholesterol and triglycerides, hypertension, cigarette smoking, obesity, and diabetes mellitus. In addition, diverse evidence highlights the role played by inflammation and clonal haematopoiesis, eventually leading to immunity involvement. The human microbiota project and subsequent studies using next-generation sequencing technology have indicated that thousands of different microbial species are present in the human gut. Disturbances in the gut microbiota (GM) composition, i.e., gut dysbiosis, have been associated with diseases ranging from localised gastrointestinal disorders to metabolic and cardiovascular illnesses. Of note, experimental studies suggested that GM, host immune cells, and marine-derived ingredients work together to ensure intestinal wall integrity. This review discusses current evidence concerning the links among GM, marine-derived ingredients, and human inflammatory disease. In detail, we summarise the impact of fish-derived proteins/peptides and algae components on CVD risk factors and gut microbiome. Furthermore, we describe the interplay among these dietary components, probiotics/prebiotics, and CVDs.

## 1. Introduction

### 1.1. Cardiovascular Disease Risk Factors

Cardiovascular diseases (CVDs), which comprise coronary heart disease, hypertension, and stroke, collectively represent the number one cause of death globally [1]. Atherosclerosis, the dominant cause of CVDs, is the process leading to the accumulation of fatty and/or fibrous material in the innermost layer of arteries, known as the intima. Subsequently, atherosclerotic plaque can acquire more fibrous material and gather calcium minerals. Advanced atherosclerotic plaques can produce a flow-limiting obstruction or disrupt and promote thrombus development. Both of these phenomena lead to tissue ischaemia and, eventually, to the clinical manifestations of atherosclerosis, i.e., myocardial infarction, stroke, and peripheral artery disease [2]. Risk factors for the development of atherosclerotic lesions and associated thrombotic complications are elevated levels of low-density lipoprotein cholesterol (LDL-C) and triglycerides (TG), hypertension, cigarette smoking, obesity, and diabetes mellitus. Diverse evidence also highlights the role played by inflammation and clonal haematopoiesis, eventually leading to immunity involvement [3,4,5].

Of note, a recent study demonstrated that in patients already taking statin therapy, the residual inflammatory risk appears to be strongly associated with future CV events rather than residual cholesterol risk [6,7]. These data strengthen the hypothesis that concomitant targeting of inflammation and atherogenic lipids will further diminish the risk of CVDs. However, conventional therapeutic approaches often exhibit limitations, i.e., side effects [8,9] and inadequate disease control [10,11,12]. Additionally, it is well known that all the risk factors for CVDs are susceptible to lifestyle modifications, such as diet and physical exercise [13]. In line with this evidence, in recent years, diverse natural products and their derivates have garnered increasing attention as an addition to “standard of care” for the treatment of CVDs [14].

### 1.2. The Microbiota–Immune Axis and CVDs: State-of-the-Art

In total, 10–100 trillion symbiotic microbial cells reside in the human body and are known as the human microbiota. The totality of these cells, belonging to bacteria, fungi, and parasites, together with genetic material (e.g., from viruses) make up the human microbiome. Our genetic ancestry is made by a combination of human and microbial species; thus, our metabolism is determined by microbial and human signatures [15].

Recent research has shown that the gut microbiota (GM) acts as an endocrine organ, playing a role in modulating immunity and influencing the development of inflammatory, metabolic, and infectious diseases [16]. When there is a balanced composition of the human microbiome, it leads to a healthy intestinal epithelial barrier and the recruitment and activation of the appropriate immune cells through the secretion of metabolites, particularly short chain fatty acids (SCFAs), and the expression of microbial components [17,18]. Furthermore, a balanced gut microbiota is crucial for the maintenance of metabolic homeostasis and the modulation of inflammatory responses, both of which are critical in the pathogenesis of CDVs. Studies have shown that alterations in GM composition (known as dysbiosis) are linked to several CVD risk factors, including hypertension, obesity, and atherosclerosis [19]. Yang et al. showed that gut dysbiosis is associated with hypertension, revealing that specific microbial profiles can influence blood pressure regulation through mechanisms involving inflammation and metabolic pathways [20]. Some studies suggest that patients with hypertension often show a lower diversified GM, along with an increased prevalence of certain bacteria, such as *Clostridiales* and *Bacteroidales* [21,22]. In the Coronary Artery Risk Development in Young Adults (CARDIA) study, systolic blood pressure was instead correlated with *Robinsoniella* and *Catabacter* abundance [23]. Furthermore, Yan et al. identified metagenomic signatures in hypertensive models, suggesting that the GM composition may serve as a biomarker for hypertension [24].

Brown and Hazen emphasised the role of microbial modulation in CVDs, suggesting that the interaction among diet, GM, and host metabolism significantly influence CVD risk [25]. This is further supported by research indicating that dietary patterns can shape the GM diversity, which in turn influences CVD outcomes [26]. Shariff et al. showed how dietary habits and interactions with the GM can lead to CV health disparities, emphasizing the relevance of understanding these relationships for effective prevention strategies [27]. This link underscores the bidirectional relationship between gut health and CV conditions, called the gut-heart axis, signifying that interventions targeting GM may have therapeutic potential for the management of CVDs [27].

The Western diet, rich in simple carbohydrates and saturated fats, along with reduced physical activity, has contributed to gut dysbiosis, altering immune balance. A shift from living in close contact with natural environments, combined with changes in eating habits, such as an increase in ultra-processed foods, red meats, and a reduction in fibre-rich foods, has led to a GM depletion [28,29]. Eventually, these conditions can lead to metabolic disorders and variations in symbiotic microorganisms, rushing the development of CVDs [30]. An increasing body of research indicates that intestinal bacteria and their metabolites are crucial in the development of CVDs. Different investigations indicating a correlation between GM and CVDs identified SCFAs and trimethylamine-N-oxide (TMAO) as crucial metabolites [19]. Specifically, SCFAs, produced through fermentation of dietary fibres, have been shown to exert protective effects against hypertension and atherosclerosis by modulating blood pressure and reducing inflammation [31]. For instance, butyrate shows beneficial effects on the host, including promoting growth and reducing inflammation in intestinal epithelial cells. In addition, butyrate plays a key role in maintaining immune balance in the gut by facilitating communication between the host and microbiota [29]. Conversely, TMAO, generated from some dietary components (e.g., choline and carnitine), has been implicated in promoting atherosclerosis development [19]. Indeed, the gut bacterial TMA lyase produces TMA from dietary choline and carnitine [32] that it is subsequently adsorbed by intestinal cells and transported to the liver. In the liver, TMA is oxidised to TMAO by the enzyme flavin-containing monooxygenase 3 (FMO3). A meta-analysis of prospective studies revealed that individuals with elevated plasma TMAO levels had a 23% higher risk of CV events [33] and a 62% greater risk of all-cause mortality [34]. Altogether, these data suggest that TMAO has been linked to an elevated CVD risk [35].

In addition, cigarette smoke may affect GM composition and function by upregulating oxidative stress-related enzymes, modifying the gut mucin layer and the expression of intestinal tight junction proteins, and stimulating the spread of non-commensal bacteria [36]. The GM of smokers differs from that of non-smokers, with a higher relative abundance of *Actinobacteria* and *Cyanobacteria* [37].

Finally, nutritional interventions targeting the GM, such as probiotics, prebiotics, and postbiotics, have shown promise in preventing CVDs, especially when implemented early in life [38].

### 1.3. Marine-Derived Compounds, GM Modulation, and CVDs

Marine species, including mammals, fish, seaweeds, sea anemones, and sponges, represent approximately one-half of the global biodiversity. Therefore, the sea offers a wonderful resource for novel compounds potentially able to improve the health of the worldwide population. Attention has been drawn to the beneficial effects of fish consumption due to the ability of fish ingredients, mainly omega-3 polyunsaturated fatty acids (n-3 PUFAs) and proteins, to lower CVD risk factors [39,40,41] and modulate inflammation [42,43,44,45].

**Figure 1:**
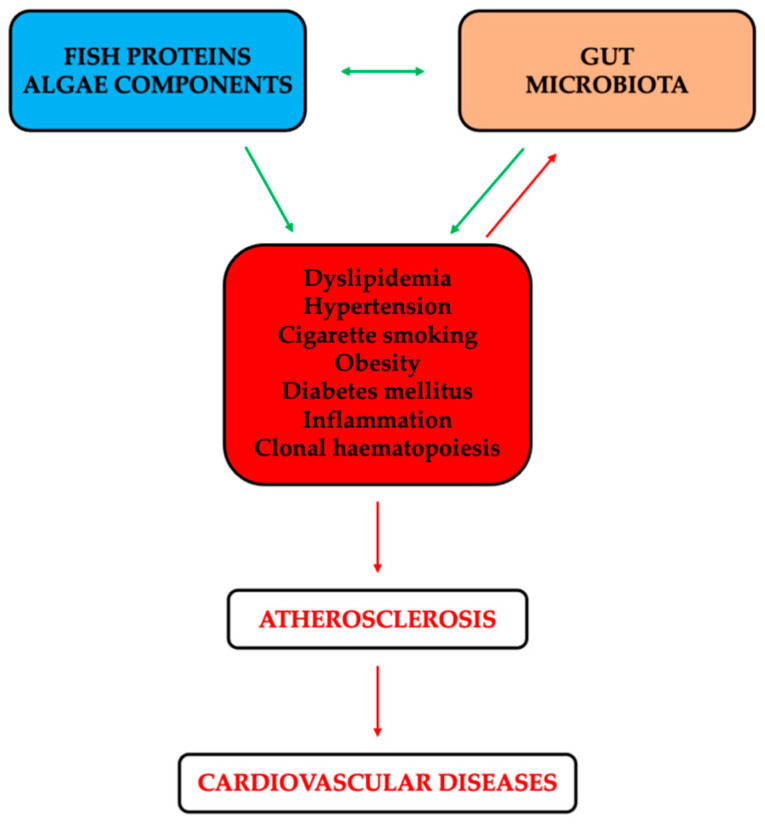
Flow chart representing the interplay among cardiovascular diseases risk factors, gut microbiota, and fish proteins/algae components. Beneficial and detrimental effects are shown by green and red lines, respectively.

Based on these data, the US Food and Drug Administration (FDA) has formally declared that the consumption of up to 3 g/d of marine-derived n-3 PUFAs is generally considered as safe. In line with this health claim, fish consumption is still recommended in the 2020–2025 Dietary Guidelines for Americans and by the American Heart Association [46]. Additionally, a recent positional paper strengthened the positive relationship between the replacement of proteins from red meat with proteins from fish and a reduced risk of CVDs [47]. In agreement, two meta-analyses reported a significant inverse association between fish consumption and all-cause mortality, with a nadir at a consumption of 60–80 g/d. In addition, this inverse association is markedly influenced by regional differences [48,49]. Moreover, in Western studies, Jayedi et al. found that the risk of all-cause and CVD mortality decreased in a dose-dependent manner and then increased with a relatively sharp trend, suggesting a U-shape curve association. While, in Asian studies, this dose-response relationship was linear [49]. Recently, Zhou et al. in a general Chinese population found a reverse J-shaped association between fish-derived protein and new-onset hypertension [50]. Thus, there is a window of consumption (appropriate level) where the risk of new-onset hypertension is lower. These data confirmed previously results that showed a U-shaped curve association between protein intake and health [51]. Additionally, recent data have documented seaweeds as promising reservoirs of bioactive compounds able at targeting multiple aspects of CVDs, including inflammation [52,53]. Specifically, seaweeds contain polysaccharides, proteins, pigments, lipids, sterols, terpenes, and phenolic compounds [53]. Additionally, the consumption of marine-derived products, especially fish proteins and compounds extracted from algae, has been increasingly recognised for their potential to modulate GM and influence CVD risk factors (Figure 1).

The mechanisms through which marine-derived compounds affect GM and CV health are multifaceted. Some studies have documented that fish-derived proteins can influence lipid metabolism and modulate the plasma lipid concentrations, contributing to lowering the CVD risk [54]. The bioactive components found in algae, such as polysaccharides and polyphenols, exhibit antioxidant and anti-inflammatory properties, that can enhance GM diversity and functionality. Specifically, polysaccharides, by acting as prebiotics, can promote the growth of beneficial gut bacteria and enhance the production of SCFAs while inhibiting pathogenic strains, thereby improving gut health and reducing systemic inflammation [55]. Polyphenols possess anti-inflammatory properties, which help maintain gut barrier integrity and prevent dysbiosis, both of which often linked to CVD risk factors [56,57]. Moreover, the antioxidant properties of marine-derived compounds can mitigate oxidative stress, a significant factor in CVD development. Indeed, by reducing oxidative stress, these compounds can help lower the risk of endothelial dysfunction and atherosclerosis development [58].

This review summarises the impact of marine-derived ingredients, including fish-derived proteins and algae components, on CVD risk factors and the gut microbiome. Furthermore, we describe the interplay among these dietary components, probiotics/prebiotics, and CVDs.

## 2. Fish-Derived Proteins/Hydrolysates/Peptides and CVD Risk Factors

Fish-derived proteins contain all the essential amino acids, mainly lysine and leucine; some non-essential amino acids (aspartic acid, glutamic acid, and alanine); and the amino acid-derived organic acid taurine [59,60,61,62]. Different studies have proved that the enzymatic digestion of fish by-products is an efficient means of producing peptides with enhanced bioactivity [61]. Furthermore, experimental and clinical data have shown the impact of fish-derived proteins/hydrolysates/peptides on lipid profile, glucose metabolism, inflammation, and blood pressure.

### 2.1. Experimental Studies

**Figure 2:**
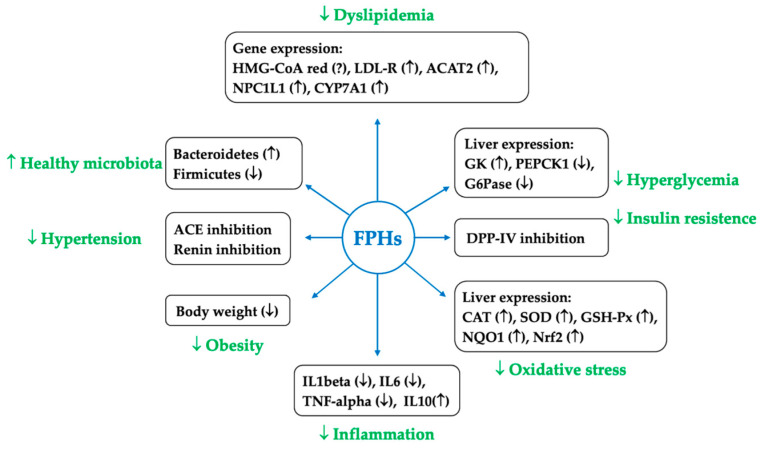
Schematic representation of the mechanisms of action of fish protein hydrolysates (FPHs). HMG-CoA red—3-hydroxy-3-methyl-glutaryl-coenzyme A reductase; LDL-R—low-density lipoprotein-receptor; ACAT2—acyl-CoA:cholesterol acyltransferase; NPC1L1—Niemann–Pick C1-like 1; CYP7A1—cholesterol 7-alpha-hydroxylase; GK—glucokinase; PEPCK1—phosphoenolpyruvate carboxikinase1; G6Pase—glucose-6-phosphate; DPP-IV—dipeptidyl peptidase-IV; CAT—catalase; SOD—superoxide dismutase; GSH-Px—glutathione peroxidase; NQO1—quinone oxidoreductase 1; Nrf2—nuclear factor-erythroid 2-related factor 2; IL—interleukin; TNF—tumour necrosis factor; ACE—angiotensin-I-converting enzyme.

A recent systematic review and meta-analysis described all the pre-clinical data performed in rodents and published before 15 July 2022 [41]. This paper concluded that the intake of proteins from fish muscles or fish by-products significantly decrease circulating total cholesterol (TC) concentrations when compared to their control group. Of note, the authors highlighted that the stronger effect of fish-derived protein intake was observed in the subgroup comprising genetically modified rodent models, which spontaneously develop hypertension after birth, and rodents fed diets enriched with cholesterol alone or in combination with cholate (added to exacerbate hypercholesterolaemia) [41,63,64,65]. These data, indicating that the potency for preventing an increase in TC concentrations was higher than that for lowering TC plasma levels, may have relevant clinical applications, albeit not directly transferable to humans. Furthermore, the authors analysed diverse mechanisms of action to justify the hypocholesterolaemic effects exerted by the dietary intake of fish or fish proteins (Figure 2).

They found that in almost half of the analysed studies, a lower TC concentration was associated with higher faecal excretion of cholesterol and/or bile acids. Furthermore, in two papers [66,67], the above-described effects were also combined with higher mRNA expression levels of cholesterol 7-alpha-hydroxylase (CYP7A1) [68], which is the first and rate-liming enzyme in cholesterol metabolism, including bile acid synthesis. On the contrary, the impact of fish or fish proteins on the expression of 3-hydroxy-3-methyl-glutaryl-coenzyme A reductase (HMG-CoA red), LDL-receptor (LDL-R), and acyl-CoA:cholesterol acyltransferase (ACAT2) was difficult to assess because expression was marked influenced by the rodent model used [41]. However, an elegant and recent work demonstrated that the hypocholesterolaemic effect exerted by Alcalase-silver carp hydrolysate (Alcalase-SCH) was associated with an up-regulation of LDL-R expression and a down-regulation of Niemann–Pick C1-like 1 (NPC1L1) and ACAT2 [69]. In addition, these authors identified novel peptides, present in the Alcalase-SCH, as main contributors to the hypocholesteroleamic activity of Alcalase-SCH [69]. Of note, in line with data obtained with soya, potato, and rice proteins, lower methionine/glycine and lysine/arginine ratios were also observed in fish proteins compared with casein, together with a lower TC plasma level. In addition, salmon protamine is a strongly alkaline polycationic low-molecular-weight protein, in which nearly two-thirds of the amino acid composition is arginine [70,71,72]. It is well known that arginine, being a precursor of nitric oxide (NO), may positively affect vascular function [73]. Indeed, arginine supplementation has been shown to decrease neointimal formation in animal models [74,75] and to improve flow-mediated vasodilation in humans [76].

These data were confirmed by different experiments conducted from July 2022 to November 2024. The findings are reported in Table 1.

In details, oral administration of jellyfish collagen hydrolysate (JCH) was able to prevent the increase in serum glucose, TC and TG levels, together with the body weight in a mouse model of obesity where mice were fed a high-fat diet (HFD) [77]. Additionally, JCH administration modulated oxidative stress and the inflammatory response, crucial factors implicated in obesity-related pathologies, and helped recover the alteration in microbiota composition induced by HFD, specifically by contrasting the lowering of *Romboutsia*’s abundance [77] (Table 1). Similar data were published by Shi et al. in healthy mice fed a chow diet and treated with half-fin anchovy hydrolysate (HAHp) or with its Maillard reaction products (HAHp-MRPs) by oral gavage [78]. Significantly, the glycation process or Maillard reaction, which is the chemical process involving proteins and sugars during food processing, can enhance protein and peptide functionalities, including antioxidant and antihypertensive activity. The glycated proteins or peptides may resist digestion and undergo fermentation in the colon, potentially benefiting gut health. Studies have shown that glycated proteins, such as those from pea [82,83] and milk, can exhibit similar probiotic effects as galactooligosaccharides (GOSs) alone [84]. GOSs are a type of prebiotic that support beneficial intestinal bacteria and produce SCFAs that have a variety of biological functions, hence promoting gut health [85]. GOSs ferment quickly, producing gas and bloating. This has raised interest in prebiotics that affect the distal colon and are linked to a lower risk of colon cancer [86]. The effect of glycated peptides on the GM remains unclear, though [87]. Jin et al. [88] investigated the effects of GOS glycated with fish peptides on GM of rats using the Maillard reaction. The composition of the gut microbiota and colonic fermentation were affected by the new glycoconjugates, offering the first in vivo proof of these prebiotic effects. Additionally, Han et al. [89] explored the chemical characteristics of glycoconjugates of myofibrillar proteins from grass carp that were conjugated with glucose via the Maillard reaction during dry heating. Glycation increased furosine levels, promoted structural changes in the proteins, and reduced protein digestibility. Butyrate production during fermentation was influenced by glycation. In addition, the amount of butyrate was directly correlated to the presence of *Mitsuokella, Lachnospiraceae_UCG-004, Sutterella, Salinimicrobium, Fodinibius*, and *Nitriliruptor* and inversely associated with the presence of *Enterococcus*, *Dorea*, *Escherichia-Shigella*, and *Phascolarctobacterium*. These findings demonstrated that the glycation of myofibrillar proteins could have positive outcomes on gut health [89].

Lin et al. found that small-molecule peptides from the bone collagen of *Harpadon nehereus* (HNCP) exerted anti-diabetic effects in mice with streptozotocin-induced diabetes [79]. Specifically, HNCP administration significantly decreased the plasma levels of glucose, TC, TG, and LDL-C and increased high-density lipoprotein (HDL)-C concentrations and insulin secretion. Moreover, HNCP improved glucose metabolism and showed remarkable antioxidant activity in this type 1 diabetic mouse model by regulating the expression levels of glycosynthases and gluconeogenesis-related [i.e., glucokinase (GK), phosphoenolpyruvate carboxykinase 1 (PEPCK1), and glucose-6-phosphate (G6Pase)] and antioxidant enzymes [i.e., catalase (CAT), superoxide dismutase (SOD), glutathione peroxidase (GSH-Px), and quinone oxidoreductase 1 (NQO1)]. Additionally, the same authors demonstrated that this latter effect, namely, the antioxidant activity, was mediated by the activation of the nuclear factor-erythroid 2-related factor 2 (Nrf2) pathway [79]. The crucial role played by Nrf2 in redox balance, inflammation, cytotoxicity, and cellular metabolism and its involvement in many oxidative stress-based diseases has been reported [90]. Similar results were obtained in streptozotocin-induced diabetes rats treated with the small peptide (<1 kDa) fraction from *Takifugu bimaculatus* skin hydrolysate (TBP) [80]. Specifically, TBP was chosen because it exhibited the strongest dipeptidyl peptidase-IV (DPP-IV) inhibitory activity in an in vitro assay. DPP-IV inhibition hinders the degradation of glucagon-like peptide 1 (GLP-1) and glucose-dependent insulinotropic polypeptide (GIP) that are released post-prandially, increasing their half-life and amplifying the insulin effect on glucose homeostasis [91]. In the in vivo experiment, TBS diminished weight loss, lowered fasting blood glucose concentrations, increased insulin’s secretion, improved irregular hormonal fluctuations and lipid metabolism, and mitigated histopathological damage in the pancreas and liver. Additionally, the relative abundance of Firmicutes decreased, alongside an increase in Bacteroidetes, and significant modifications were observed at the genus level. In addition, two metabolites, hippuric acid and ergosta-5,7,22,24(28)-tetraen-3beta-al, were identified following TBP administration [80]. In line with these data, a salmon peptide fraction (SPF) containing low-molecular-weight peptides was able to prevent the development of obesity and metabolic disorders, dampening inflammation in both hepatic and intestinal tissues, and modulate thrombosis risk factors in high-fat and high-sugar-fed vitamin D-deficient dyslipidaemic mice [81]. Interestingly, Fang et al. applied a multistage strategy, in detail, a molecular docking-based virtual screening using a small library of marine-derived natural products with follow-up in vitro and in vivo phenotypic assays, aiming at discovery new lipid-lowering molecules [92].

Hypertension, as already mentioned above, is a major risk factor for CVD. One pharmacological approach aiming at reducing blood pressure is represented by the angiotensin-I-converting enzyme (ACE) inhibitors. ACE is a key enzyme that catalyses the conversion of angiotensin I (an inactive decapeptide) to angiotensin II (octapeptide), a potent vasoconstrictor, which stimulates the release of aldosterone, and eventually increases blood pressure. Fish-derived bioactive peptides have been widely investigated for their anti-hypertensive effects, such as ACE inhibition. All the studies published before 2020 have been collected in reviews [40,93,94]. According to the results obtained, the bioefficacy and bioavailability of the final peptide products are markedly affected by the extraction processes used (the enzymatic hydrolysis as well as isolation/purification techniques). Additionally, size and chain length together with the presence of some amino acids (tyrosine, tryptophan, proline, and phenylalanine) at the C-terminal end of the fish-derived peptide structures are crucial for ACE inhibition and antihypertensive effects [40,93,94]. In vitro experiments demonstrated that protein hydrolysates from fish by-products exerted competitive, non-competitive, and mixed inhibition modes against ACE.

Recently, Shao et al. [95], using in silico, molecular docking, and in vitro analysis, isolated hydrolysates six novel peptides that possess potent ACE inhibitory and antioxidant activities from *Sardina pilchardus* (Table 2).

These six peptides (Phe-Ile-Gly-Arg, Gly-Ile-Leu-Arg, Phe-Gln-Arg-Leu, Phe-Arg-Ala-Leu, Lys-Phe-Leu, and Lys-Leu-Phe) exert zinc-chelating capacity, as confirmed by molecular docking and competitive inhibition. Similar results were observed in two studies with hydrolysate peptides derived from Monkfish (*Lophius litulon*) swim bladders, the latter being a by-product inducing pollution issues. The first one used papain digestion of the whole swim bladders and identified eighteen antioxidant peptides. Among these, three peptides (Tyr-Asp-Tyr-Asp, Ala-Arg-Trp, and Asp-Asp-Gly-Gly-Lys) exhibited the highest ability for radical scavenging, lipid peroxidation inhibition, and protective function against oxidation-induced DNA damage [96]. In the second study, Hu et al. separated three peptides from pepsin-soluble swim bladder collagen of Monkfish (*Lophius litulon*) with marked hypotensive effects [97]. Moreover, these authors demonstrated, using an in vitro model, that these peptides, identified as Ser-Glu-Gly-Lys, Phe-Asp-Gly-Pro-Tyr, and Ser-Pro-Gly-Pro-Trp, exerted their hypotensive activities throughout a combination of ACE inhibition and a modulation of the production of NO and endothelin-1 (ET-1) [97]. Finally, the same authors characterised different peptides from protein hydrolysate of skipjack tuna (*Katsuwonus pelamis*) milts [98,105] and from muscle hydrolysate of Miiuy croaker [99,106], which possess the above-mentioned activities, as demonstrated by molecular docking and in vitro studies.

In line with these data, in vivo experiments, mainly performed in spontaneously hypertensive rats (SHRs), proved the strong antihypertensive activity of protein hydrolysate from diverse marine organisms (Table 2). Of note, grass carp peptides, rich in phenylalanine, leucine, aspartic acid, and glycine, significantly reduced systolic blood pressure compared to the control group treated with captopril, the drug of choice for hypertensive patients [93]. Similar data were obtained by Chen et al. when administering the Leu-Ser-Gly-Tyr-Gly-Pro peptide from Nile tilapia (*Orechromis niloticus*) skin gelatine to SHRs [100,107]. Moreover, these authors used molecular docking comparisons and identified four connecting residues of the ACE active site, which may justify the mechanism of inhibition [100]. Recently, an experimental study demonstrated that both intact and hydrolysed blue whiting proteins reduced blood pressure in an obese rat model, inhibiting renin activity but not showing ACE inhibitory effects [101]. A peptide composed of 13 amino acid residues, Asp-Pro-Ala-Leu-Ala-Thr-Glu-Pro-Asp-Pro-Met-Pro-Phe, obtained from Nile tilapia (*Orechromis niloticus*) exhibited potent ACE inhibitory and radical scavenging activities, suggesting its potential use in functional foods [102]. Indeed, the administration of Zebra blenny protein hydrolysates to rats fed a high-cholesterol/cholic acid-containing diet attenuated cholesterol-caused cardiac injury, as demonstrated by biochemical and histological improvements as well as significantly protection of heart genomic DNA from oxidative damage induced by Fenton’s reagent [103]. Finally, Maneesai et al. investigated the impact of tuna protein hydrolysate (TPH) on CV remodelling and dysfunction in a rat model of metabolic syndrome [104]. The results of this study demonstrated that TPH supplementation improved all the metabolic parameters, including dyslipidaemia, hyperglycaemia, obesity, hypertension, cardiac hypertension, endothelial dysfunction, oxidative stress, and inflammation, in a dose-dependent manner. These effects were related to TPH’s ability to modulate angiotensin II receptor type 1 (AT_1_R)/NADPH oxidase 2 (NOX2), endothelial nitric oxide (eNOS), Nfr2/haem oxygenase 1 (HO-1) and peroxisome proliferator-activated receptor (PPAR)gamma/nuclear factor kappa B (NF-kB) protein expression in heart and aortic tissues [104].

Various experimental studies have investigated the impact of fish protein hydrolysates (FPHs) from salmon or anchovy by-products (spine, viscera, collagen) on atherosclerosis development. All the studies were performed on genetically modified mice, including apoE-deficient mice, mice fed a high-fat diet [108,109] or mice fed a high-fat/high-cholesterol diet [110,111]. Altogether, the results demonstrated that these FPHs reduced plaque area and lipid accumulation in the aorta as well as in the aortic sinus. Conversely, no differences in extracellular matrix, macrophages, and T lymphocytes were observed in the plaque area of FPH-fed mice compared to control animals. Of note, these effects were associated with lower levels of pro-inflammatory cytokines in the serum and aorta [108,109,110,111,112]. Interestingly, two studies showed that taurine proved efficacious in reducing the development of atherosclerosis in apoE-deficient mice fed a chow diet with or without TMAO [113,114]. Furthermore, the authors demonstrated that dietary taurine exerted its anti-atherosclerotic effects via increasing the hepatic gene expression of conjugated bile acid synthesis and, eventually, increasing the conjugated BA to unconjugated BA ratio in the liver as well as serum. Meanwhile, taurine improved the TMAO-induced abnormal bile acid profile in the gallbladder. Moreover, taurine increased bile acid deconjugation by enhancing the levels of the genus *Ruminiclostridium* level and the excretion of faecal neutral sterols. In line with the data obtained with the FPHs, taurine positively modified TMAO-induced inflammation in both serum and the aorta [113,114].

Altogether, these in vitro and in vivo experimental studies highlighted the crucial interplay among fish-derived proteins/hydrolysates/peptides, CVD risk factors, and the GM milieu. Therefore, it is plausible to speculate that these effects could be easily translated to humans. However, different factors should be considered by researchers before developing a clinical protocol. First of all, all the in vitro data have to be confirmed in diverse experimental models. Conversely, additional in vivo experiments should be performed to better reproduce the human condition, aiming at finding the therapeutical range for dose administration as well as treatment time.

### 2.2. Clinical Studies

Diverse clinical studies investigating the impact of fish-derived peptides on CVD risk factors have been performed thus far. Three reviews summarised the clinical trials published before 2020 [39,61,93]. Additionally, a recent systematic review and meta-analysis of randomised controlled trials (RCTs) investigated the impact of lean fish and fish-derived protein consumption on the lipid profile [115]. Even though some of the studies reported an overall positive metabolic effect of consuming different fish protein hydrolysates, such as effects on body weight [116,117], TG and TC concentrations [118,119,120], glucose metabolism [121,122,123,124], and hypertension [125], the majority of RCTs show highly inconsistent results. Indeed, Tou et al. concluded that additional well-designed, longer, and larger RCTs are mandatory to achieve a final statement of the impact of lean fish and fish proteins on serum lipid levels [115]. Moreover, these new clinical studies are needed to appropriately inform the public about nutritional differences among fish species, eventually helping consumers to make more evidence-based dietary choices [115]. In line with the above-citated experimental data, more clinical studies documented the beneficial activity of taurine on CVD risk factors [62]. Notably, taurine seems to reduce blood pressure by acting as an antagonist of angiotensin II [126].

## 3. Seaweed Components and CVD Risk Factors

Different studies have shown beneficial effects of seaweed polyphenols, especially florotannins, on inflammation, oxidative stress, hyperglycaemia, and hyperlipidaemia [127]. Additionally, fucoxanthin and brown seaweed-derived florotannins have significant anti-inflammatory and antioxidant activities, which may contribute to CV protection [128]. Moreover, marine microalgae provide vital nutrients and metabolites, including carotenoids and polysaccharides, which may help prevent heart disease [129]. By including these substances in functional foods, the worldwide burden of CVDs may be reduced, and CV health may be improved. Overall, despite ongoing clinical application challenges, the range of pharmacological activity of marine compounds offers a unique possibility for novel CVD treatments [130].

### 3.1. Experimental Studies

Algal polysaccharides, particularly fucoidan and laminarin, have attracted attention for their therapeutic potential in atherosclerosis. In vitro studies have shown that algal polysaccharides can inhibit LDL oxidation, a critical step in the development of atherosclerosis. This antioxidant effect is essential for preventing the formation of foam cells, which contribute to plaque formation in the arteries [131]. In addition, the observed anti-inflammatory properties of these polysaccharides may help at reducing vascular inflammation, eventually supporting CV health. Moreover, these compounds have been shown to inhibit atherosclerotic plaque formation and improve endothelial function [131].

Studies in genetically modified mice demonstrated the role of fucoidan (a polysaccharide composed of L-fucose extracted from brown seaweed) in atherosclerosis management [132,133] (Table 3).

Specifically, these studies showed that intragastric gavage or intraperitoneal administration of fucoidan proved efficacious in decreasing arterial plaque formation as well as macrophage plaque accumulation and smooth muscle cell proliferation (Table 1). In addition, the authors reported the ability of fucoidan to improve the lipid profile based on reductions in TC, LDL-C, and TG and an increase in HDL-C. Looking for the potential mechanisms of action in fucoidan-treated mice, they found that the hepatic gene expression of sterol regulatory element-binding protein (SREBP)1 (SREBP), acetyl-CoA carboxylase (ACC), fatty acid synthetase (FAS), SREBP2, and HMG-CoA reductase was down-regulated, whereas LDL-R gene expression was up-regulated compared with control animals [132,133]. In conclusion, these data suggest that fucoidan is able to impair atherosclerotic plaque development by increasing lipid metabolism/uptake and decreasing lipid synthesis. Finally, they also reported a reduction in reactive oxidative species (ROS) as well as of pro-inflammatory mediators. In other words, these compounds could be used as dietary supplements for the atherosclerosis prevention and management.

The anti-obesity action of *Eisenia bicyclis* (*Kjellman*) Setchell (EEB) 30% ethanol extract has been tested on 3T3-L1 preadipocytes and C57BL/6 mice fed a HFD [134]. 3T3-L1 cell differentiation, proliferation, and mitotic clonal expansion (MCE) were all lowered by EEB treatment. In the subcutaneous and liver tissues of HFD-fed mice, oral treatment of EEB inhibited lipogenesis and adipogenesis. EEB increased thermogenesis in brown adipose tissue (BAT) [134]. Mice given oral doses of *Monostroma nitidum* rhamnan sulphate (RS) showed a considerable rise in body weight and food intake, accompanied by a decrease in plasma TC, glucose, and insulin levels. This latter effect testifies that RS improves insulin resistance. RS feeding modified the GM by activating pathways linked to glycolysis and the tricarboxylic acid cycle (TCA) and reducing the *Firmicutes*/*Bacteroidetes* (F/B) ratio, thus exerting an anti-obesity effect [135].

In mice fed a high-fat/high-cholesterol diet, the serum levels of TC, LDL-C, and TG were significantly lowered, and the expression of NPC1L1, a crucial transporter for intestinal cholesterol absorption was downregulated, by laminarin treatment [136].

Oligosaccharides extracted from *Enteromorpha prolifera* oligosaccharides (EPOs) manifested antioxidative, anti-inflammatory, and anti-diabetic effects when administrated to mice [137]. EPOs regulate the crotonylation of XPO1 (exportin for nuclear export of NES-containing proteins and RNAs) and HSPA8 (heat shock protein family A member 8) proteins, modulating the expression of key genes involved in cell cycle and aging. EPOs are also involved in glucose metabolism by inhibiting the crotonylation of HSPA8-K126 and activating the AKT pathway. Finally, EPOs promote the crotonylation of histones in intestinal cells, increasing the abundance of the butyric acid-producing bacteria *Ruminococcaceae* [137]. In a diabetic mouse model, *Enteromorpha prolifera* sulphated polysaccharides (EPs) enhanced glucose tolerance, decreased blood glucose levels, and boosted liver glycogen content. The ability of EPs to boost AKT phosphorylation was found to be responsible for its anti-diabetic benefits. This, in turn, led to reductions in glycogen synthase kinase-3β (GSK-3β) and Forkhead box protein O1 (FOXO1) activity, which in turn encouraged glycogen production and reduced gluconeogenesis [119]. In addition, EPs inhibited the elevated expression of O-GlcNAc transferase (OGT) induced by diabetes, suggesting that EPs act similarly to an OGT inhibitor and contributed to mitigate hyperglycaemia [138].

The three *Undaria pinnatifida* extracts, including *Undaria pinnatifida* low-temperature water extract (UPLW), *Undaria pinnatifida* high-temperature water extract (UPHW), and UPE, *Undaria pinnatifida* ethanol extract, had different chemical profiles, suggesting that the extraction approach had an impact on the extracts’ compositions [146]. UPLW exhibited the highest inhibitory impact on sucrose, but UPHW was more effective in inhibiting α-glucosidase activity toward maltose. After oral delivery of glucose, maltose, or sucrose to mice, the UPLW extract was the most successful in lowering postprandial blood glucose levels in those animals [146].

A recent review by Fernando et al. [147] describes the methodologies to obtain protein and protein hydrolysates from microalgae, and the studies demonstrated their biological properties. The nutritional relevance of the microalgae is due to their high protein content, ranging from 50 to 70%, based on species, growth phase, and light quality [148]. Similar to what was mentioned earlier for FPHs, these microalgae-derived proteins, mainly their bioactive peptides obtained through hydrolysis by proteolytic enzymes or microorganisms, display antioxidant, anti-inflammatory, antihypertensive, and immunomodulatory effects, which are crucial in reducing CVD risk factors. Antioxidant peptides have been produced by the hydrolysis of *Chlorella* sp. [139], *Navicula* sp. [140], and *Spiurulina* sp. [141] and extensively investigated in in vitro experiments. However, more studies are need to identify the amino acid sequences with antioxidant capacity and then to verify these data in vivo models as well as clinical trials. Antihypertensive peptides derived from *Chlorella vulgaris*, *Chlorella ellipsiodea*, *Spirulina platensis,* and *Nannochloropsis oculate* have been tested using both in vitro and in vivo (mainly using SHRs) experimental settings [142,143]. The most potent antihypertensive peptides seem to contain a high percentage of hydrophobic amino acid residues, such as proline, and the basic mechanisms are related to ACE and renin inhibitions [128]. Finally, two in vitro studies demonstrated the capacity of peptides isolated from *Chlorella pyrenoidosa* and *Spirulina maxima* to down-regulate the gene expression levels of E- and P-selectins, intercellular cell adhesion molecule-1 (ICAM-1), vascular cell adhesion molecule-1 (VCAM-1), monocyte chemoattractant protein (MCP-1), and ET-1 [144,145].

Although the potential CVD efficacy of proteins produced from algae appear promising, more in vivo experimental studies are required to confirm these results. Finally, clinical trials are needed to validate the pre-clinical data and to investigate the potential applications of these proteins in functional foods and nutraceuticals [149,150].

### 3.2. Clinical Studies

According to a review, polyphenol-rich marine extracts significantly reduced TC, LDL-C, and glucose plasma levels, but no relevant effects were observed on other biomarkers related to CVDs, such as postprandial glucose and fasting insulin plasma concentrations, indicating the need for further research to clarify these relationships [151].

Paradis et al. [152] investigated the effect of brown seaweeds (*Ascophyllum nodosum* and *Fucus vesiculosus*) on glucose and insulin responses after carbohydrate intake in a double-blind, placebo-controlled trial with 23 participants. A single dose (500 mg) of seaweed reduced insulin levels by 12.1% and increased insulin sensitivity by 7.9%; however, it did not significantly impact glucose levels (Table 4).

Lee and Jeon [153] examined the effects of 12 weeks of daily supplementation with a dieckol-rich extract (AG-dieckol) from *Ecklonia cava* on glycaemic control in 80 pre-diabetic adults. Compared to placebo, the AG-dieckol group showed significant reductions in post-meal glucose, insulin, and C-peptide levels. No adverse effects or abnormal changes in blood parameters were observed. These results suggest AG-dieckol and similar phlorotannin-rich marine algae may help manage post-meal blood sugar and insulin sensitivity, potentially benefiting diabetes treatment.

Regarding hypertension, a pilot study explored the effects of *Spirulina maxima* on 16 patients treated with ACE inhibitors over 12 weeks. The authors showed significant decreases in systolic blood pressure and endothelial damage markers (VCAM-1, E-selectin, and ET-1) and increases in antioxidant activity (glutathione peroxidase and oxidised glutathione) [154].

A form of green microalgae called *Chlorella* has also been the subject of several clinical studies examining its impact on CV risk factors [155,156,159]. A systematic review found that supplementation with *Chlorella* can reduce fasting blood glucose, TC, and LDL-C concentrations and systolic and diastolic blood pressure, but no effects were observed on TG and HDL-C levels. *Chlorella*’s rich nutritional profile, which includes antioxidants and phytochemicals that may act in concert to support CV health, is believed to be the source of its health benefits [159].

Regarding metabolic syndrome, one study showed the potential beneficial effects of the algae compound Gdue [157]. Gdue is an algal extract made from the combination of *Ascophyllum nodosum* and *Fucus vesiculosus*, together with chromium picolinate. The two algae that make up Gdue have a 95:5 ratio (*Ascophyllum nodosum* 95, *Fucus vesiculosus* 5), and help maintain body weight and promote metabolism, especially the metabolism of fats and carbohydrates [157]. Specifically, overweight or obese patients with elevated fasting LDL-C levels were treated with Gdue for six months. Gdue-treated patients showed improvements in metabolic syndrome metrics. These included reductions in body weight and waist circumference, blood pressure, and fasting blood TG, LDL-C, and glucose concentrations. These effects were associated with a drop in HbA1c and a rise in HDL-C levels. Overall, the study showed a 27.7% relative reduction in CVD risk [157].

Finally, ProAlgaZyme is a novel and proprietary infusion of freshwater algae in purified water. The effects of the infusion on the CV risk factors associated with metabolic syndrome were assessed in a randomised double-blind placebo-controlled study with 60 overweight and obese individuals aged 25 to 60 [158]. Over the course of ten weeks, ProAlgaZyme consumption significantly improved serum lipid profiles, reduced inflammatory markers, and significantly decreased weight and glucose levels in overweight and obese subjects [158]. According to a recent review [151], marine polyphenols found in algae possess anti-inflammatory properties that may help at reducing the inflammatory responses, eventually improving the CVD profile.

## 4. Beneficial Effects of Probiotic/Prebiotics and Marine-Derived Compounds

Due to potential health benefits, especially regarding the GM and CVDs, marine-derived ingredients have stimulated a bunch of investigations. Additionally, marine sources are a rich source of prebiotics, which are non-digestible food components that specifically promote the growth and activity of a healthy gut microbiota.

Probiotics are able to reduce TMAO concentrations, with *Lactobacillus rhamnosus GG* showing efficacy in both animal and human studies. However, the probiotics’ effects on TMAO reduction appear to be strain specific [160]. Furthermore probiotics, prebiotics, and synbiotics have demonstrated efficacy in lowering cholesterol concentrations, mitigating inflammation and exhibiting antioxidative and antiplatelet effects [161]. Both probiotics and prebiotics can modify the GM composition, promoting the proliferation of beneficial bacterial strains and rectifying dysbiosis linked to CVD risk factors [162].

Additionally, faecal microbiota transplantation has emerged as a potential therapeutic approach for CVDs [163]. Altogether, these data underscore the relevance of gut microbiota-targeted strategies in CVD prevention and treatment. Nevertheless, despite encouraging findings from in vitro studies, animal research, and select human clinical trials, additional rigorously designed clinical investigations are imperative to comprehensively elucidate the long-term effects and underlying mechanisms of these dietary interventions in the context of CVD prevention and treatment.

Recent studies have placed a great deal of effort in studying the function of fish-derived proteins and how they affect the human microbiome. In detail, one study documents that dietary protein sources, including fish hydrolysates, can alter GM composition and enhance beneficial bacteria [26]. Importantly, FPHs possess excellent digestibility, absorption, water-holding capacity, texture, gelling, whipping, and emulsification properties when introduced into the food matrix [164].

Gabolysat^®^, a fish protein hydrolysate extracted from cod and mackerel, known for having anxiolytic [165] and gastric protective effects [166], may exert beneficial actions on the colonic mucosal barrier integrity, especially by increasing the mRNA levels of the anti-inflammatory cytokine interleukin (IL) 10 [167]. Fish protein hydrolysates, such as those from salmon and mackerel, have been shown to increase beneficial bacteria while reducing harmful strains in mice GM, suggesting a protective role against metabolic diseases [168]. In addition, Sivixay et al. demonstrated that the combination of fish proteins and the prebiotic raffinose positively affects the GM composition and its metabolic functions. Specifically, the data evidenced that this dietary combination influenced GM diversity by increasing the abundance of *Akkermansia muciniphila* [169]. This bacterium is the most abundant species in the human intestinal microbiota, and it has been inversely associated with body weight, inflammatory index, insulin resistance, glucose tolerance, and the development of atherosclerosis in several experimental studies [170,171].

Marine algae polysaccharides (MAPs) have the potential to modify the gut microbiota in a way that improves heart failure treatment and CV health. MAPs can stimulate GM to produce healthier SCFAs. By influencing the gut microbiome’s regulation of bile acid metabolism, MAPs can also improve CV health [172]. According to de Brito Alves et al. [173], supplementation with *Spirulina platensis* increases the production of beneficial microbial metabolites like SCFAs, improves gut barrier function, and enhances the GM diversity and composition, which, as previously reported, has been linked to a number of health advantages, including anti-inflammatory, anti-obesity, and anti-diabetic effects. To evaluate *Spirulina platensis*’s impact directly on the gut microbiome, more clinical trials are still needed.

## 5. Material and Methods

The usage of the MeSH tool in PubMed allowed us to browse through NLM databases. Using this tool, we were able to refine our search and emphasise the relevant studies. The following search terms were combined: “fish proteins” OR “fish protein hydrolysate” OR “fish peptides” OR “algae” OR “seaweed” OR “anchovy” OR “blue whiting” OR “bonito” OR “carp” OR “catfish” OR “cod” OR “flatfish” OR “haddock” OR “halibut” OR “herring” OR “mackerel” OR “Miiuy Croaker” OR “Monkfish” OR “pollock” OR “redfish” OR “saithe” OR “salmon” OR “sardine” OR “Sardina” OR “sprat” OR “saury” OR “tilapia” OR “trout” OR “tuna” OR “turbot” AND “mice” OR “mouse” OR “rat” OR “rodent” OR “clinical studies” OR “human studies” AND “cholesterol” OR “bile acid” OR “hypercholesterolemia” OR “LDL” OR “triglycerides” OR “HDL” OR “atherosclerosis” OR “diabetes” OR “hypertension” AND “gut microbiota” OR “microbiota composition” OR “gut microbiota diversity”. The search was updated until September 2024 during the first submission and until November 2024 during the revision process.

## 6. Conclusions

CVDs represent the number one cause of death globally, and atherosclerosis is the dominant cause of CVDs. Risk factors for the development of atherosclerotic lesion are dyslipidaemia, hypertension, cigarette smoking, obesity, and diabetes mellitus. Diverse evidence also highlights the role played by inflammation and clonal haematopoiesis, eventually leading to immunity involvement. Of note, it is projected that the overall direct medical costs associated with CVDs will experience a dramatic increase, reaching USD 818 billion. In addition, the indirect expenses, a consequence of the decreased productivity, will rise up to USD 276 billion. Therefore, there is an urgent need for affordable and efficacious preventive and therapeutic strategies for individual at high risk of or diagnosed with CVDs [162]. The GM acts as an endocrine organ, playing a role in modulating immunity and influencing the development of inflammatory, metabolic, and infectious diseases. In fact, gut dysbiosis has been associated with inflammatory-based pathologies ranging from localised gastrointestinal disorders to metabolic and CV illnesses. There is growing evidence that FPHs and algae compounds have positive effects on the CV system. In addition, including these marine-derived ingredients in dietary choices is a viable way to improve both the CVD risk factors and GM health, eventually leading to a lower risk of the clinical manifestation of atherosclerosis (Figure 1). Prebiotics and probiotics work synergistically to promote gut health, which is essential for preserving CV health. However, although the potential health advantages of combining fish-derived proteins/hydrolysates/peptides or algae components with prebiotics/probiotics seem promising, further specific experimental and human studies are needed to validate the above-mentioned beneficial effects and, notably, safety, aiming at comprehensively comprehending their clinical implications. Furthermore, speaking about prebiotics/probiotics, it is crucial to keep in mind that the impact of these substances can be influenced by different factors, such as the strains used, the quantity and the duration of the supplementation, as well as the individual’s GM composition and overall health condition. Tailored clinical trials including patients in primary and secondary CV prevention strategies are mandatory to design new therapeutic protocols, where the “standard of care” will be implemented with these combinations of fish- and algae-derived ingredients and prebiotics/probiotics, aiming at decreasing the side effects of traditional drugs and, hopefully, the patient’s compliance [8]. Finally, the results of these future well-designed clinical trials are welcomed by companies to formulate new functional foods and nutraceuticals.

## Figures and Tables

**Table 1:** Fish-derived proteins/hydrolysates/peptides showing effects on CVD risk factors in experimental studies.

Fish Name	Component	CVD Model	Effects	Ref.
Jelly fish	Collagen hydrolysate	Mice, high-fat diet	Hypoglycaemic,	[77]
			hypolipidaemic,	
			body weight reduction,	
			modulation of oxidative stress and	
			inflammatory response, recovery of	
			gut microbiota diversity	
Half-fin anchovy (*Setipinna taty*)	Muscle hydrolysate	Mice, chow diet	Hypoglycaemic,	[78]
			hypolipidaemic,	
			modulation of oxidative stress	
			and inflammatory response	
*Harpadon nehereus*	Bone collagen peptides	Mice, streptozotocin-induced diabetes	Hypoglycaemic,	[79]
			hypolipidaemic,	
			increased insulin secretion and antioxidant activity	
*Takifugu bimaculatus*	Skin hydrolysate small peptides	Rats, streptozotocin-induced diabetes	DPP-IV inhibitory activity,	[80]
			hypoglycaemic,	
			hypolipidaemic,	
			body weight reduction,	
			increased insulin secretion,	
			pancreas and liver damage decrease,	
			recovery of gut microbiota diversity	
Salmon	Frame small peptides	Mice, high-fat and high-sugar diet	Hypoglycaemic,	[81]
			hypolipidaemic,	
			antithrombotic,	
			modulation of liver and	
			intestinal inflammation	

DPP-IV—dipeptidyl peptidase-IV.

**Table 2:** Fish-derived proteins/hydrolysates/peptides showing antihypertensive effects.

Fish Name	Peptide	Model	Effects	Ref.
*Sardina pilchardus*	Phe-Ile-Gly-Arg, Gly-Ile-Leu-Arg, Phe-Gln-Arg-Leu, Phe-Arg-Ala-Leu, Lys-Phe-Leu, Lys-Leu-Phe	In vitro	Antihypertensive, antioxidant	[95]
Monkfish (*Lophius litulon*)	Tyr-Asp-Tyr-Asp, Ala-Arg-Trp, Asp-Asp-Gly-Gly-Lys	In vitro	Antihypertensive, radical scavenging, lipid peroxidation inhibition, protective function against oxidation-induced DNA damage	[96]
Monkfish (*Lophius litulon*)	Ser-Glu-Gly-Lys, Phe-Asp-Gly-Pro-Tyr, Ser-Pro-Gly-Pro-Trp	In vitro	Antihypertensive, radical scavenging, lipid peroxidation inhibition, protective function against oxidation-induced DNA damage	[97]
Skipjack tuna (*Katsuwonus pelamis*)	Hydrolysates	In vitro	Antihypertensive,	[98]
			radical scavenging,	
			lipid peroxidation inhibition,	
			protective function against	
			oxidation-induced DNA damage	
Miiuy Croaker	Hydrolysate	In vitro	Antihypertensive,	[99]
			radical scavenging,	
			lipid peroxidation inhibition,	
			protective function against	
			oxidation-induced DNA damage	
Grass carp	Hydrolysates	In vivo (SHR)	Antihypertensive	[93]
Nile tilapia (*Orechromis niloticus*)	Leu-Ser-Gly-Tyr-Gly-Pro	In vitro, in vivo (SHR)	Antihypertensive	[100]
Blue whiting	Proteins, Hydrolysates	In vivo (obese rats)	Antihypertensive	[101]
Nile tilapia (*Orechromis niloticus*)	Asp-Pro-Ala-Leu-Ala-Thr-Glu-Pro-Asp-Pro-Met-Pro-Phe	In vitro	Antihypertensive, radical scavenging	[102]
Zebra blenny	Hydrolysates	Rats fed a high-fat/cholic containing diet	Biochemical and histological improvement in cardiac tissue, protective function against oxidation-induced DNA damage	[103]
*Tuna*	Hydrolysate	Rats fed a high-fat diet	Hypoglycaemic; Hypolipidaemic; Antihypertensive; modulation of oxidative stress, endothelial dysfunction, and inflammatory responses	[104]

SHR—spontaneously hypertensive rats.

**Table 3:** Algae-derived components showing effects on CVD risk factors in pre-clinical studies.

Compound	Source	Model	Effects	Mechanisms	Ref.
Fucoidan	Brown seaweeds	In vivo	Reduction in atherosclerotic plaque development, macrophage accumulation, and smooth muscle cell proliferation	Downregulation of SREBP1, ACC, FAS, SREBP2 and HMG-CoA reductase; upregulation of LDL-R gene expression	[132,133]
Ethanol extract	*Eisenia bicyclis* (*Kjellman*)	In vitro, in vivo (mice fed a high-fat diet)	Anti-obesity	Inhibits lipogenesis/adipogenesis; increases thermogenesis in brown adipose tissue	[134]
Sulphated polysaccharide	*Monostroma nitidum*	In vivo (mice fed a high-fat diet)	Hypoglycaemic, hypolipidaemic, increased insulin secretion	Improves gut microbiota diversity	[135]
Laminarin	Brown algae	In vivo (mice fed a high-fat/high-cholesterol diet)	Reduced TC, LDL-C, TG	Downregulates NPC1L1	[136]
Oligosaccharide	*Enteromorpha prolifera*	In vivo	Antioxidant, anti-inflammatory, hypoglycaemic and anti-diabetic	Modulation of the expression of proteins related to cell cycle and aging, AKT pathway regulation, increases the butyric acid-producing bacteria *Ruminococcaceae*	[137]
Sulphated polysaccharide	*Enteromorpha prolifera*	In vivo	Hypoglycaemic, anti-diabetic	Modulation of AKT, GSK-3β, FOXO1,and OGT expression	[138]
UPLW, UPHW, UPE	*Undaria pinnatifida*	In vivo, mice	Hypoglycaemic	Inhibition of α-glucosidase	[137]
Peptides	*Chlorella* sp., *Navicula* sp., *Spirulina* sp.	In vitro, in vivo (SHRs)	Antioxidant, anti-inflammatory, antihypertensive	ACE and renin inhibition, downregulation of ICAM-1, VCAM-1, MCP-1, and ET-1 gene expression	[139,140,141,142,143,144,145]

SREBP—sterol regulatory element-binding protein; ACC—acetyl-CoA carboxylase; FAS—fatty acid synthetase; HMG-CoA red—3-hydroxy-3-methyl-glutaryl-coenzyme A reductase; LDL-R—low-density lipoprotein-receptor; NPC1L1—Niemann–Pick C1-like 1; GSK-3β—Glycogen synthase kinase-3β; FOXO1—Forkhead box protein O1; OGT—GlcNAc transferase; SHR—spontaneously hypertensive rats; ACE—angiotensin-I-converting enzyme; ICAM-1—intercellular cell adhesion molecule-1; VCAM-1—vascular cell adhesion molecule-1; MCP-1—monocyte chemoattractant protein; ET-1—endothelin-1.

**Table 4:** Algae-derived components showing effects on CVD risk factors in clinical trials.

Compound	Source	Effects	Study Type	Ref.
Polyphenols	*Ascophyllum nodosum*, *Fucus vesiculosus*	Hypoglycaemic	Randomised crossover placebo-controlled	[152]
Dieckol-rich extract	*Ecklonia cava*	Hypoglycaemic	Double-blind, randomised, placebo-controlled	[153]
Whole	*Spirulina maxima*	Antihypertensive	Exploratory placebo-controlled	[154]
Whole	*Chlorella vulgaris*, *Chlorella pyrenoidosa*	Hypoglycaemic, hypolipidaemic, antihypertensive	Randomised placebo-controlled	[155]
Whole	*Chlorella vulgaris*	Hypolipidaemic	Double-blinded, randomised, placebo-controlled	[156]
Whole	*Ascophyllum nodosum*, *Fucus vesiculosus*	Hypoglycaemic, hypolipidaemic, antihypertensive	Observational	[157]
Whole	Algae	Reduction in weight, body, hypoglycaemic, hypolipidaemic, modulation of inflammatory response	Randomised double-blind, placebo-controlled	[158]

## Data Availability

No new data were generated.

## Abbreviations

CVDs	cardiovascular diseases
LDL-C	low-density lipoprotein cholesterol
TG	triglycerides
GM	gut microbiota
SCFAs	short-chain fatty acids
TMAO	trimethylamine-N-oxide
FMO3	flavin-containing monooxygenase 3
FDA	Food and Drug Administration
n-3 PUFAs	omega-3 polyunsaturated fatty acids
TC	total cholesterol
FPHs	fish protein hydrolysates
HMG-CoA red	3-hydroxy-3-methyl-glutaryl-coenzyme A reductase
LDL-R	low-density lipoprotein-receptor
ACAT2	acyl-CoA:cholesterol acyltransferase
NPC1L1	Niemann–Pick C1-like 1
CYP7A1	cholesterol 7-alpha-hydroxylase
GK	glucokinase
PEPCK1	phosphoenolpyruvate carboxikinase1
G6Pase	glucose-6-phosphate
DPP-IV	dipeptidyl peptidase-IV
CAT	catalase
SOD	superoxide dismutase
GSH-Px	glutathione peroxidase
NQO1	quinone oxidoreductase 1
Nrf2	nuclear factor-erythroid 2-related factor 2
IL	interleukin
TNF	tumour necrosis factor
ACE	angiotensin-I-converting enzyme
NO	nitric oxide
GOSs	galactooligosaccharides
HDL-C	high-density lipoprotein-cholesterol
GLP-1	glucagon-like peptide 1
GIP	glucose-dependent insulinotropic polypeptide
SHRs	spontaneously hypertensive rats
ET-1	endothelin-1 (ET-1)
AT_1_R	angiotensin II receptor type 1
NOX2	NADPH oxidase 2
eNOS	endothelial nitric oxide
HO-1	haem oxygenase 1
PPAR	peroxisome proliferator-activated receptor
NF-kB	nuclear factor kappa B
RCTs	randomised controlled trials
SREBP	sterol regulatory element-binding protein
ACC	acetyl-CoA carboxylase
FAS	fatty acid synthetase
GSK-3β	glycogen synthase kinase-3β
FOXO1	Forkhead box protein O1
OGT	O-GlcNAc transferase
ICAM-1	intercellular cell adhesion molecule-1
VCAM-1	vascular cell adhesion molecule-1
MPC-1	monocyte chemoattractant protein
XPO1	exportin for nuclear export of NES-containing proteins and RNAs
HSPA8	heat shock protein family A member 8
